# Performance of a Novel Frameless and Maskless Robotic Head Motion Compensation System for Stereotactic Radiosurgery in a Realistic Clinical Environment with Healthy Volunteers

**DOI:** 10.1016/j.ijrobp.2025.09.029

**Published:** 2025-09-23

**Authors:** Xinmin Liu, Ahmad Sakaamini, Wenbo Gu, Carl Denis, Michelle Alonso-Basanta, Rodney D. Wiersma

**Affiliations:** a Department of Radiation Oncology, University of California, Los Angeles, California; b Department of Radiation Oncology, University of Pennsylvania, Philadelphia, Pennsylvania; c CDR Systems, Calgary, Alberta, Canada

## Abstract

**Purpose:**

Stereotactic radiosurgery (SRS) is a nonsurgical method for treating brain abnormalities and small tumors. Traditional high-accuracy SRS requires a rigid metal head frame screwed into the skull, which causes discomfort and reduces patient compliance. Thermoplastic masks offer a less invasive alternative but compromise accuracy because of flexing and are often still uncomfortable. To address these issues, we developed a novel robotic head motion compensation (RHMC) device that enables frameless and maskless SRS.

**Methods and Materials:**

A compact, portable RHMC device was developed that can be quickly attached to or detached from the end of a linear accelerator treatment table. Real-time 6° head position tracking was performed using 3-dimensional surface-guided radiation therapy imaging, which was fed into the robot control computer. Device performance was evaluated by administering virtual SRS treatments to a phantom and 20 healthy volunteers, simulating a clinical environment but without delivering radiation. The primary success metric was defined as maintaining the 6D target position under a 1.0 mm and 1.0° threshold for more than 95% of beam-on time (denoted as DC95%_1.0 mm and 1.0°).

**Results:**

Two of the 20 volunteers were excluded because of incompatibility with the RHMC device. Among the remaining 18 volunteers, the DC95%_1.0 mm and 1.0° success metric was achieved in all cases. Without the RHMC device, 9 of the 18 volunteers were able to meet this metric. For a tighter tolerance of DC95%_1.0 mm and 0.5°, 17 volunteers achieved the metric with the RHMC device, compared with 4 without. For a tolerance of 1.0 mm and 1.0°, across all 18 volunteers, the mean and range were 99% and 96% to 100% using the RHMC device, respectively, compared with 73% and 9% to 100% without the RHMC device.

**Conclusions:**

The RHMC device effectively maintained accurate head motion control under simulated clinical conditions, achieving the DC95%_1.0 mm and 1.0° success metric for all suitable candidates. This technology has the potential to enable frameless and maskless SRS delivery within or better than current standard-of-care tolerance guidelines.

## Introduction

Stereotactic radiosurgery (SRS) is a form of radiation therapy (RT) used for the treatment of brain disorders using highly precise radiation dose placement. Unlike conventional open brain surgery, SRS allows access to sites that would otherwise be difficult or inadvisable to treat because of potential surgical complications to the brain stem, arteries, and other vital structures. The most common treatment site for SRS has been benign or metastatic brain tumors, which account for 30% to 50% of all SRS cases in medical centers.^1^ The Central Brain Tumor Registry of the United States reported a total of 467,894 cases of primary brain and other central nervous system tumors from 2017 to 2021, with an average of 93,578 cases per year.^2^ Randomized studies have demonstrated that SRS significantly improves disease-free survival, reduces neurologic complications, and potentially improves the overall survival of certain subsets of patients with brain metastases relative to whole-brain RT.^3–5^ Additionally, SRS is increasingly used to treat arteriovenous malformations, trigeminal neuralgia, and acoustic neuromas.^6–10^

To protect critical neural and vascular structures in the brain, SRS requires a steep radiation dose falloff around target volumes. This requirement is fulfilled through greater mechanical and radiation dose and patient positioning accuracy than that of conventional RT. To achieve the American Association of Physicists in Medicine Task Group #42 recommendation of an overall targeting error of <2 mm for intracranial SRS,^11^ head immobilization frames or thermoplastic masks are used to lock patients’ heads into a precise 3-dimensional (3D) stereotactic position, suppressing voluntary and involuntary physiological movement. The discomfort, inconvenience, and invasive nature of frame preparation have been identified as major causes of poor patient compliance and clinical efficiency when SRS is medically indicated.^12^ For certain patients with extreme cranial anatomy or existing surgical bone flaps, ring placement is not possible. In addition, frames cannot be used when a hypo-fractionated schedule is desired, leading to the use of less accurate techniques. Eliminating the frame by the use of thermoplastic face masks reduces the accuracy of SRS, because mask flex can lead to systematic drift away from the target.^13^ Additionally, the accuracy of mask-based immobilization depends strongly on mask quality and the skill of the person applying the mask and is affected by mask shrinkage during treatment and physical changes to the patient’s head because of swelling or weight loss. It has been reported that many patients find thermoplastic masks to be invasive and psychologically confining, because they greatly restrict natural head motion, and mask placement is impossible for some patients with claustrophobia.^14,15^ All of these factors can create unsafe conditions, resulting in treatment interruption to realign patients and ensure target coverage.^16^

Research into the utilization of the robotic head motion compensation (RHMC) device during SRS provides notable advantages for patients, because it eliminates the need for frame or mask use.^17–19^ The central principle underlying the RHMC device is the maintenance of a patient’s head in a position close to the desired fixed 3D position when the patient is relaxed and supine, with the head supported by a 6°-of-freedom (6DoF) platform. Continuous submillimeter and subdegree positional adjustments are made to the platform are made to compensate for involuntary physiological movement. Because the platform is coupled loosely to the patient’s head and attached flexibly to the neck, corrections made by the robot in a particular direction effectively translate to head position corrections in the same direction. This method improves not only patient comfort but also the accuracy of radiation delivery, because prior feasibility studies have shown the potential of reducing head motion to under a 0.5 mm and 0.5° threshold 99% of the time.^20^ Here, parallel-kinematic Stewart platforms, or hexapod robots, which enable full 6DoF head motion correction, were used.^20^ Although adequate for proof-of-concept, such prior RHMC devices were not suitable for clinical SRS because the robot’s location was directly below the patient’s head, which prevents radiation delivery to the back of the head because of gantry collision issues. In addition, infrared (IR) tracking of external markers attached to the patient’s head was used for the real-time monitoring of head motion in these RHMC device studies. There are several potential issues associated with the rigid attachment of these markers to the head. For example, dental bite blocks have reproducibility issues during insertion and removal, are susceptible to mandibular motion, and are not appropriate for patients lacking suitable upper dentition.^21^

To address these issues, we presented the first clinically compatible frameless and maskless RHMC system for SRS. The system’s hexapod robot is located under the patient’s shoulders, allowing full linear accelerator (Linac) gantry clearance and 360° radiation beam access as required for SRS. A 3D surface-guided RT (SGRT) is used for real-time 6DoF head motion tracking, eliminating the need for external IR marker placement.

## Methods and Materials

### Robot design

**Fig. 1.**
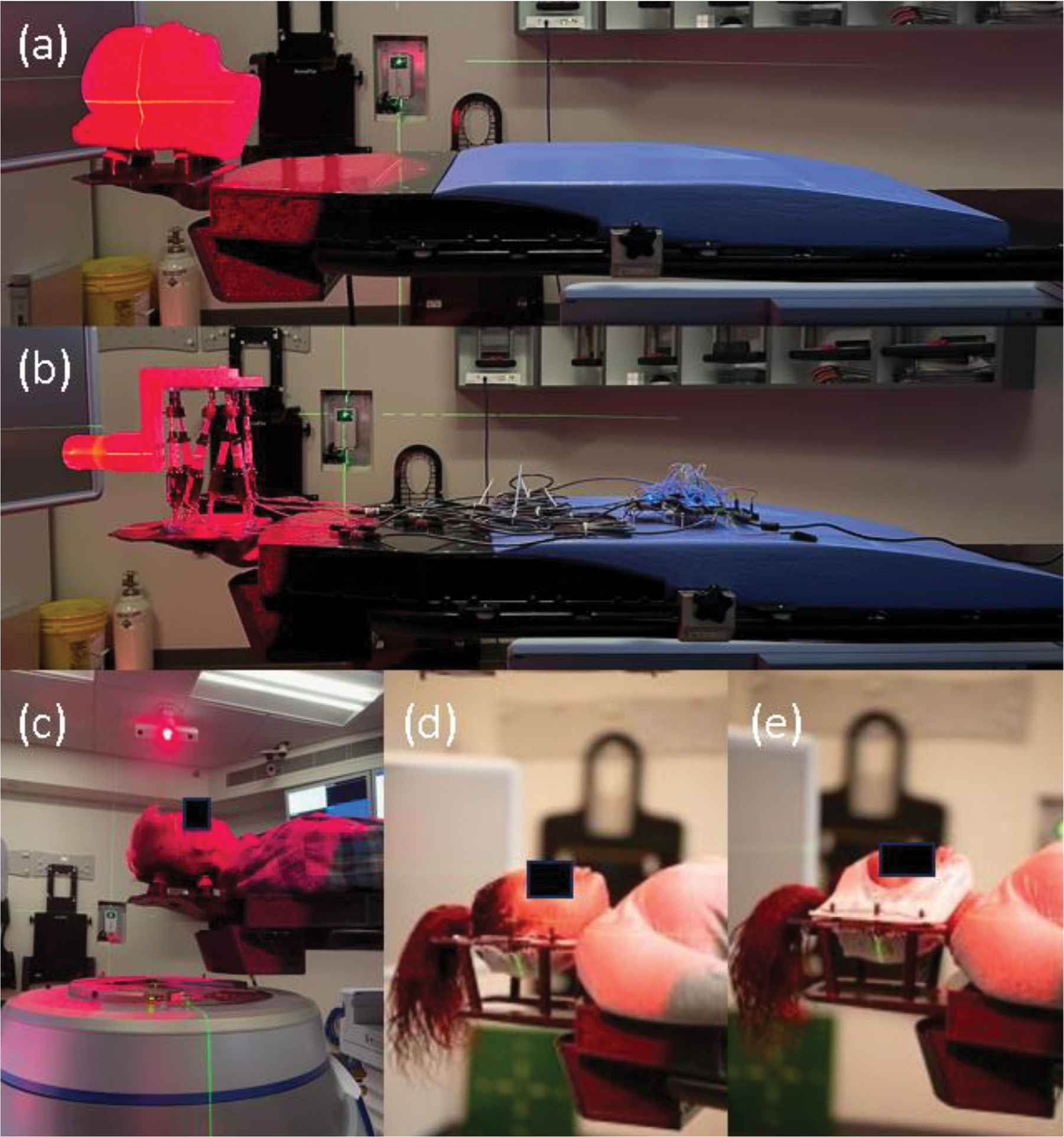
Robotic head motion compensation (RHMC) system overview for frameless and maskless stereotactic radiosurgery (SRS). (a) A compact hexapod robot located below the patient’s shoulders is used to move a head holder in 6° for real-time head motion corrections. (b) A dynamic 6° phantom used to simulate head motion. (c) Demonstration of full gantry and couch clearance for a 360° SRS HyperArc treatment. (d, e) The Head holder can be swapped for a traditional mask-based system if desired.

The RHMC system was built as a lightweight portable accessory that can be quickly attached to and detached from the end of a Linac treatment couch (Fig. 1). For head position control, a 6DoF hexapod robot with 6 linear actuators driven by NEMA 14 stepper motors was used. The robot’s maximum range of motion was limited to ±10 mm translation and ±3° rotation, and its maximum velocity was limited to 1 mm/s. To provide unobstructed 360° beam access by the Linac and to avoid collision risks, the compact robot was located directly below the patient’s shoulder blades, with a carbon fiber arm extending out to support the head and neck area. Figure 1c illustrates the 360° rotational freedom of the gantry around a volunteer, enabling SRS delivery even with the Linac isocenter placed on the individual’s frontal lobe.

To avoid treatment beam attenuation, the head holder and all other components in the beam path were made of low-atomic-density carbon fiber and foam materials. The head holder has 4 posts to ensure that it provides the required traction under the weight of the patient’s head for the effective translation of robot motions to target corrections (Fig. 1c). For comfort, the posts were made of hard foam with soft foam caps. Once the RHMC device is securely installed on the couch, the system’s relative position remains fixed, requiring no additional calibration and allowing it to function as a specialized head support. Minor adjustments to the installation position do not impact the control accuracy.

**Fig. 2.**
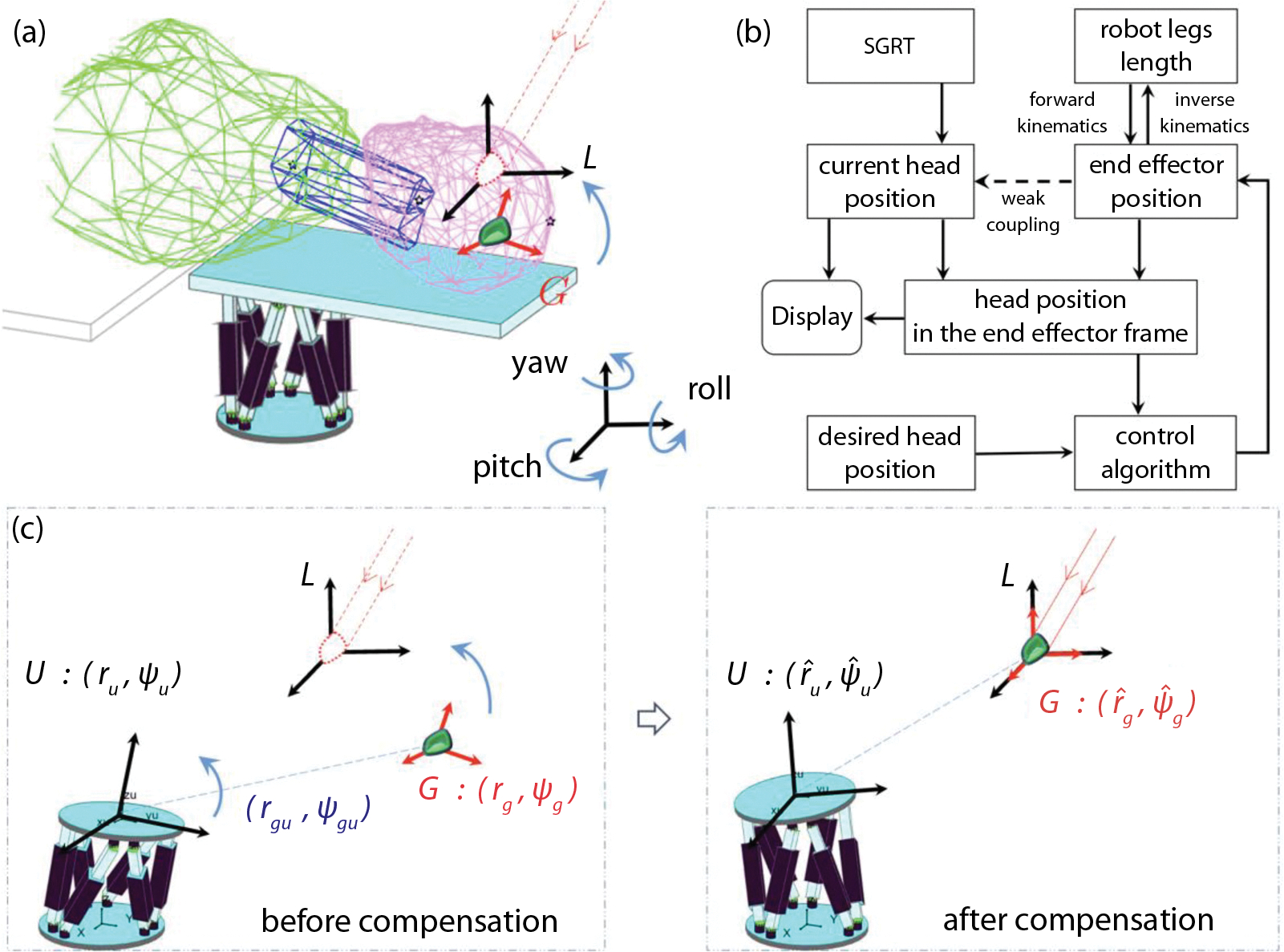
(a) Dynamic computer simulation of the robotic head motion compensation (RHMC) device. The desired clinical target position is Linac isocenter L, and the real-time camera tracked clinical target is G. (b) The objective is to move G to L by control of the robot using a closed-loop system where the camera continuously measures the target’s position, and a robot is used to move along a trajectory to maintain the target at the desired position. (c) Example before and after robot motion position to make a pitch-based clinical target correction.

The algorithm used for real-time head motion correction takes the measured 6DoF cranial motion from a real-time tracking camera and rapidly calculates a trajectory for each linear actuator to translationally and rotationally compensate the motion (Fig. 2).^20^ The clinical target in the head (*G*) can be considered as a rigid body that the robot can move along any desired trajectory in 6 dimensions (6D), which includes *x*, *y*, *z*, pitch, roll, and yaw trajectory (Fig. 2a). The desired target setpoint (*L*) corresponds to the Linac isocenter. The position of the end-effector (head support) is then represented by 6D coordinates, which were converted to coordinates in the Linac frame. The camera tracks the target position in this frame ($r_{g},\;\varphi_{g}$) in real time during treatment. The iterative control algorithm responds to measured 6DoF head positional errors in small steps without predictive modeling, as required to account for and correct the non-rigid robot-head coupling (Fig. 2b). Based on the accuracy and noise of the system’s tracking camera, a 0.2 mm, 0.2° correction threshold was selected to prevent the robot from making unnecessary corrections because of camera noise. Figure 2c shows a before-and-after example of robot leg motion for purely pitch-based clinical target correction. Additionally, the pure rotation of the clinical target around the Linac vertical axis can be accomplished with the arc-like movement of the robot in the coronal plane.

### Real-time 6D intracranial target tracking

Real-time 6D intracranial tracking of a tumor target position was performed using a commercial SGRT system (AlignRT, Vision RT).^22–24^ The submillimeter, subdegree accuracy of the SGRT camera during such tracking has been verified in numerous clinical studies.^18,24–28^ The primary advantage of SGRT tracking is that it avoids the use of external IR markers attached to the patient’s face. The entire SGRT coordinate frame was calibrated to the Linac frame such that the origin was at the radiation isocenter. The facial surface was captured and registered to the reference surface using fast iterative closest point calculation. Because the vector between the reference surface and the target is determined during patient setup, the 6D target position in the Linac frame can be calculated. This process is performed at approximately 10 frames per second (fps), but the frequency can vary depending on the size of the region of interest.^25^ Because the response time of the robot motor controller to head position updates from the SGRT camera is <1 ms, the primary lag time (potentially > 100 ms) is governed using the camera.^29^ The network User Datagram Protocol (UDP) was used for camera-controller communication.

### Phantom measurements: human head motion simulation

Before testing on human subjects, it was necessary to perform phantom experiments to verify the proper operation and performance of the RHMC device. To simulate real-time head motion, a lightweight and compact 6D robotic motion phantom was constructed and placed on the RHMC device head holder (Fig. 1b). Previously recorded volunteer head motion data from an earlier study was then input into the robotic phantom to reproduce realistic motion.^30^ To stress-test the RHMC device, we selected a motion recording with more extreme displacements—up to 6 mm and 2° deviation along certain axes. To allow unobstructed 360° Linac rotation around the phantom for SRS delivery, a specialized phantom holder was 3D printed to attach to the top of the robot and extend toward the isocenter.

### System testing with healthy volunteers

To test the RHMC system and collect head motion data for the development of head stabilization methods for brain radiation, a trial (institutional review board #843058) was conducted on 20 healthy volunteers. To simulate the clinical SRS environment, a virtual SRS treatment (HyperArc RT) was performed based on previously used clinical treatment plans. Each volunteer was asked to enter the Linac room and lie on the couch in a relaxed supine position with his or her head supported by the RHMC device head holder (Fig. 1c). The couch was then moved to position the isocenter at a midbrain target, defined using the in-room lasers. After the volunteer had had a few minutes to settle on the head holder and verbally confirmed that they were comfortable, the SGRT device was used to capture the reference surface. For each volunteer, mock SRS delivery was performed twice, with and without the RHMC device, in random order. The volunteer was not instructed or informed about the RHMC device activation. For each volunteer, one couch angle was chosen from 3 standard HyperArc couch angles: 0° (no rotation), 45°, or 90°, and tracking was done for 6 minutes. To provide a quantitative endpoint reflecting system effectiveness, the recorded 6D target motion data were analyzed using metrics similar to those used in previous studies,^17,19,30^ and positional deviation histograms showing the total percentage of time that the target remained below a specific translational and rotational tolerance level were generated. At the end of each session, the volunteer was asked to provide feedback on the overall comfort of the device.

Success was defined as maintaining the intracranial target within <1.0 mm translationally and <1.0° rotationally for more than 95% of beam-on time, expressed as the Linac duty cycle (DC). For compactness and clarity, we denote this metric as DC95%_1.0 mm and 1.0°. As described in prior studies, the RHMC device operates under the principle that when motion exceeds tolerance, fast automated beam gating is triggered by the monitoring camera to prevent radiation delivery outside tolerance.^17,19,30^ Such automated position-based beam gating is common in RT and can be done in sub-second times.^29^ Because the robot continues correcting head motion regardless of beam-on/off status, the beam is automatically re-enabled once the robot brings the head position back within tolerance. Typically, the robot can return the patient to tolerance within a few seconds, resulting in a minimal treatment delay. The 95% threshold was therefore chosen as a 5% increase of a 15-minute total SRS treatment time (~45 seconds) because the additional delay would not severely impact standard clinical workflow. For the shorter 6-minute mock SRS deliveries used in this study, the additional beam-hold time would be proportionally smaller.

The institutional review board determined that the protocol presented no risk to volunteers. The optical camera tracking system operated passively, the robot’s maximum range of motion was limited to a few millimeters in any direction, and the platform translation speed was less than 1 mm/s, thereby eliminating the risk of injury from rapid head motion. Furthermore, the volunteer’s head was not rigidly fixed to the platform; thus, if the robot’s motion exceeded the head’s natural range, the head would slide harmlessly on the holder until the motors reached their mechanical limits.

## Results

**Fig. 3.**
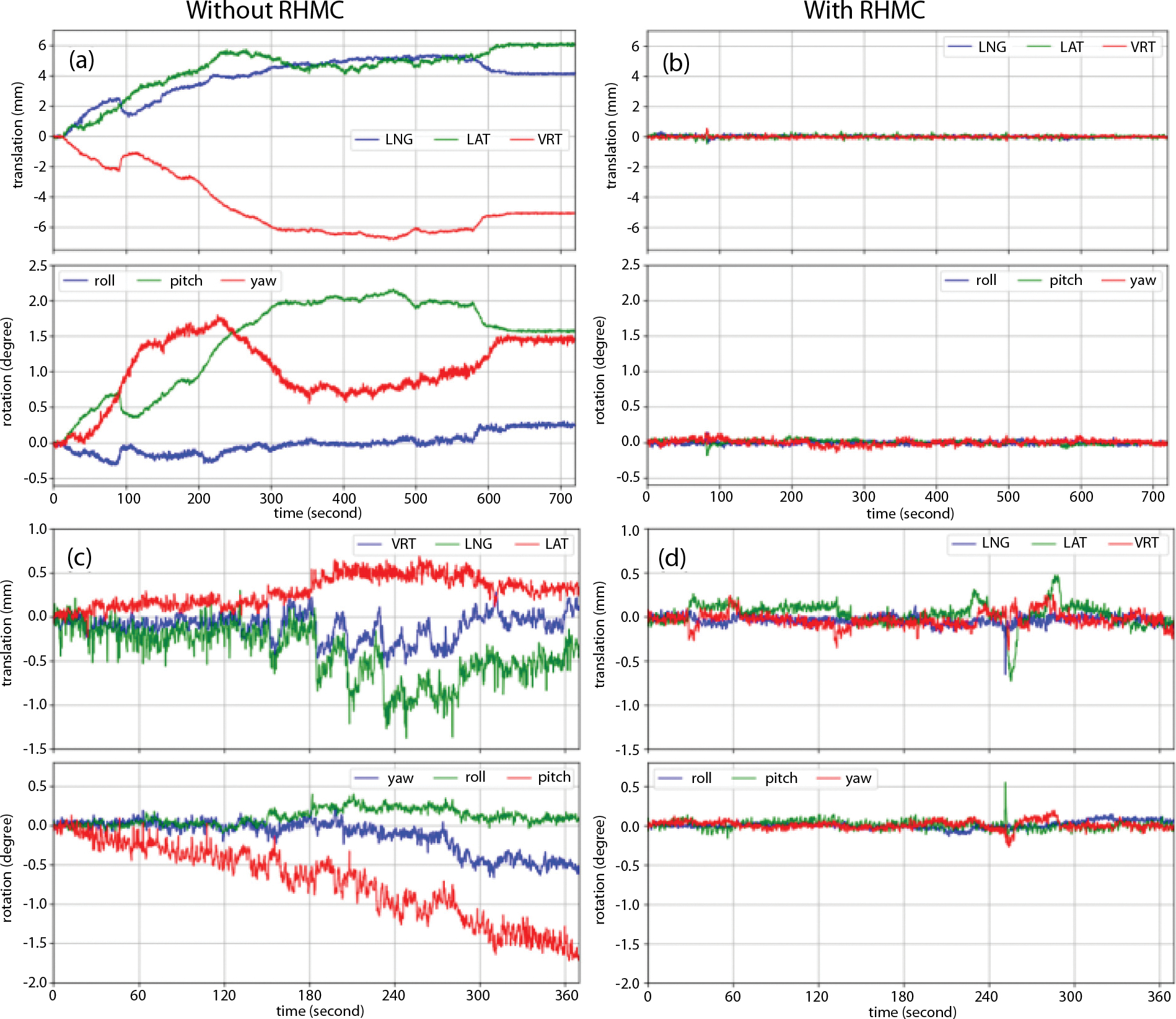
Use of a 6°-of-freedom (6DoF) dynamic head phantom to mimic real-time 6° head motion during stereotactic radiosurgery (SRS) radiation delivery. Surface-guided radiation therapy (SGRT) was used to track head motion without (a) and with the robotic head motion compensation (RHMC) device (b). Measured SGRT output for volunteer #2 is shown, comparing head motion without (c) and with the RHMC device (d).

The SGRT camera tracked the robotic head phantom over 12 minutes, both with and without the RHMC device (Fig. 3). Without the RHMC device, the phantom head gradually drifted away from the clinical target position, exceeding several millimeters and/or degrees by the end of the virtual treatment session. It remained within the 1.0 mm and 1.0° tolerance threshold less than 20% of the time. With the RHMC device, the phantom remained within the 1.0 mm and 1.0° tolerance for 99% of the time, thereby demonstrating proper operation and performance of the RHMC device and confirming readiness for subsequent human subject testing.

**Fig. 4.**
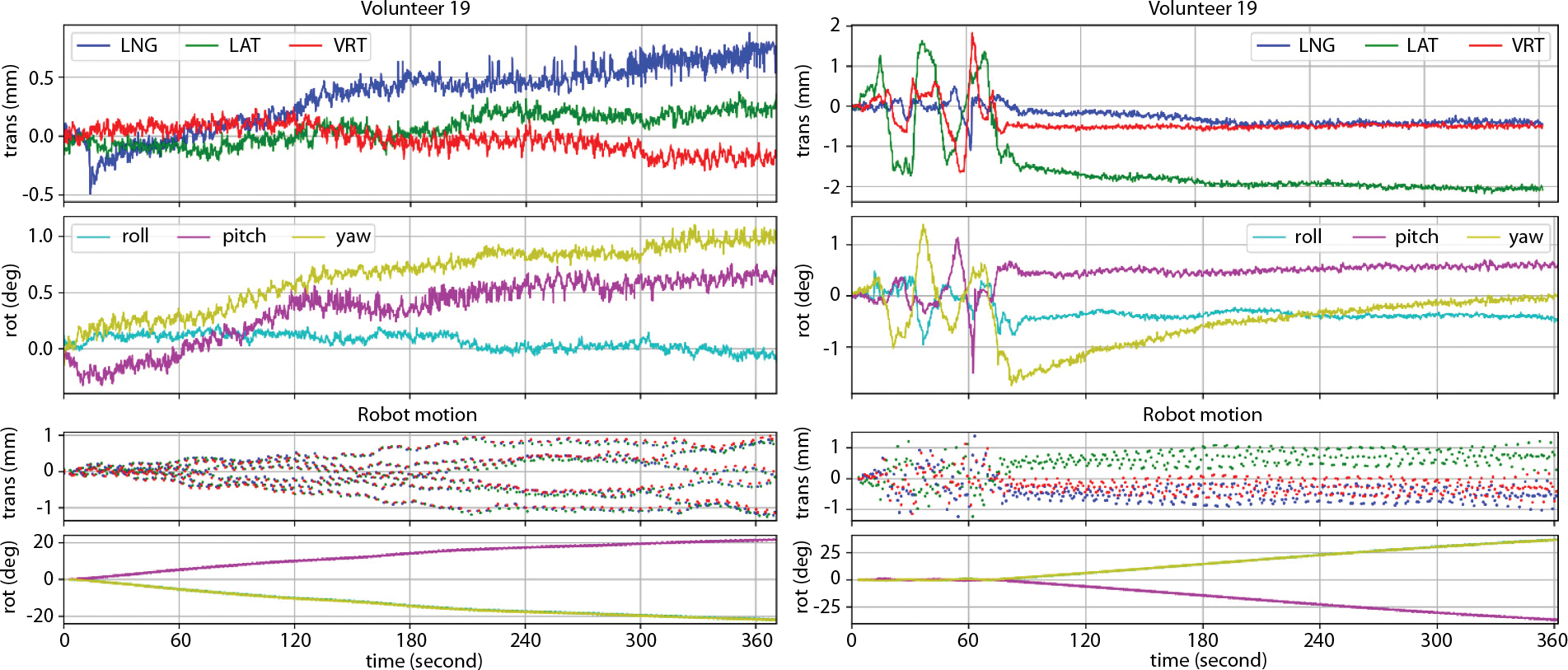
Robotic head motion compensation (RHMC) device failure cases. Volunteer #19 (left) had a smaller head size, which did not properly fit the head holder, resulting in poor head-to-robot motion coupling. Volunteer #20 (right column) had a prior neck injury, which limited neck motion in certain directions.

System performance was then assessed with 20 volunteers, monitoring head motion over a simulated HyperArc treatment duration of 6 minutes with and without the RHMC device. A midbrain target was chosen for each volunteer using the in-room lasers, and a reference surface was captured using the SGRT device. For all cases, it was verified that the gantry was able to rotate 360° around the volunteers without collision. In general, it was found that the RHMC device substantially improved the maintenance of the volunteers’ heads at the clinical target position (Fig. 3). However, for 2 volunteers, the RHMC device was unable to maintain stable positioning. One of these volunteers had a prior neck injury that prevented robot-driven neck movement in certain directions. In the other case, the single-size head support did not fit the volunteer’s head well, leading to poor coupling; the robot attempted to correct the head position until it met its range of motion limit (Fig. 4). No volunteer expressed discomfort related to the device.

**Fig. 5.**
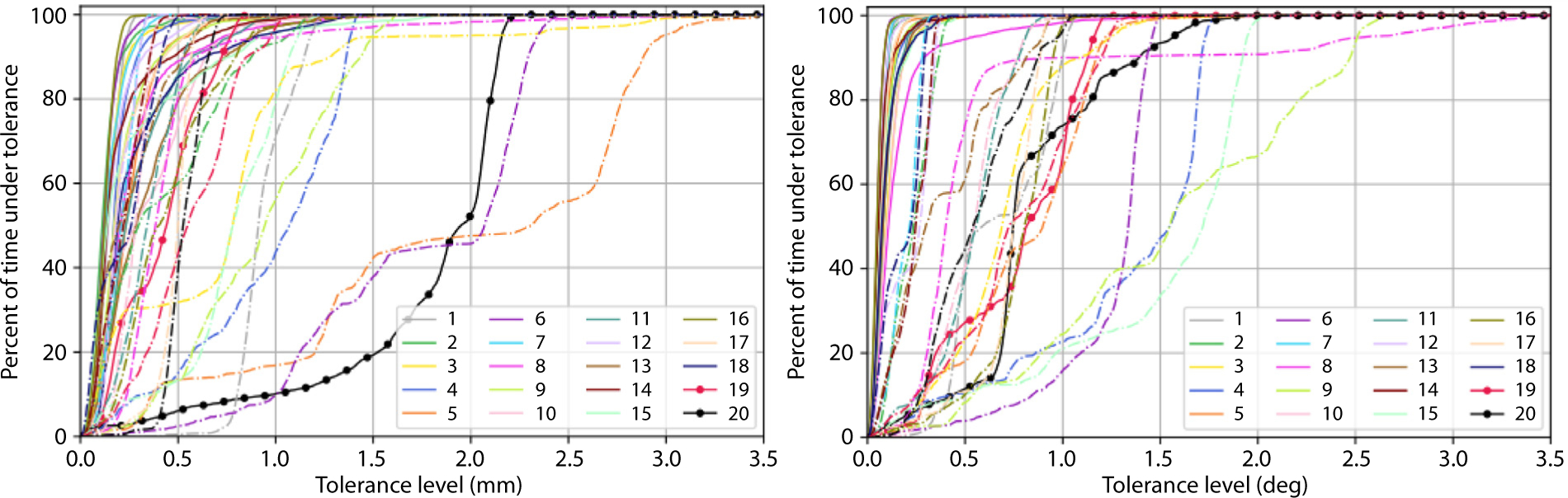
Cumulative distribution plots showing the percent of time that a healthy volunteer remained under the motion thresholds defined on the horizontal axis for a 6-minute virtual HyperArc treatment. Color represents a particular volunteer and compares the robotically corrected motion (solid lines) with the uncorrected motion (dashed lines). The 2 outlier cases are shown as solid lines with dots.

Figure 5 presents cumulative distribution plots showing the percentage of treatment time that each volunteer’s head motion remained within a given threshold. In these plots, lines positioned higher indicate better maintenance of head position within tolerance throughout the treatment duration. The clustering of solid lines near the top of the graph reflects the consistently high performance achieved with the RHMC device across most volunteers, whereas the scattered and lower-positioned dashed lines illustrate the greater variability and reduced stability observed without RHMC. The 2 outlier cases correspond to the lower end of the RHMC device range and are directly linked to identifiable mechanical or anatomic limitations rather than random performance failure.

Table 1 is a summary of Figure 5 across all volunteers. For the 18 suitable volunteers, the DC95%_1.0 mm and 1.0° success metric was achieved in all cases. Without the RHMC device, 9 of the 18 volunteers were able to meet this metric. With the RHMC device, the 1.0 mm and 1.0° tolerance had a DC of mean 99% and a range of 96% to 100%, whereas, without the RHMC device, the mean was 73%, and a broad range of 9% to 100% was seen. For the tighter tolerance of DC95%_1.0 mm and 0.5°, 17 of 18 volunteers achieved this metric with the RHMC device, compared with 4 without. Although some volunteers were able to maintain a static position very well without the RHMC device, particularly at the 1.0 mm and 1.0° level, this good performance without the RHMC device was not consistent across the cohort.

## Discussion

To our knowledge, the RHMC system tested in this study is the first of its kind and the only device potentially enabling frameless and maskless SRS delivery while remaining below recommended intervention tolerance levels.^31^ It was developed to increase the availability of SRS for patients for whom immobilization frames and masks are intolerable or uncomfortable. It also improves on the current clinical SRS workflow, as customized masks do not need to be created or applied during patient setup. Although there is the potential that real-time head motion compensation may be implemented using a 6DoF treatment couch or radiation beam compensation, it remains unclear whether such devices can perform real-time head position stabilization as they support heavy loads (ie, a patient or compact Linac), which limits their ability to make rapid kinematic responses to random real-time head position changes because of high inertia.

The current standard of care for open face-mask SRS involves manual beam deactivation and intervention when a patient’s position exceeds a predefined tolerance threshold.^32,33^ Although manual beam deactivation could be used with the RHMC device, the SGRT camera used in this study has a US Food and Drug Administration-approved automated beam shut-off feature with subsecond speed.^29^ The activation of this feature for the RHMC device would further automate SRS delivery while maintaining the patient’s target head position. Because the robot responds quickly, the beam could be reactivated automatically as soon as the robot moves the patient back into the tolerance zone following the occurrence of possible beam-gating scenarios, which could be triggered by coughing, swallowing, sitting up, panic attacks, sneezing, or other situations where involuntary or voluntary motion may occur.^34^ In this way, treatment interruption for manual intervention would be reduced or eliminated, and the current SGRT SRS workflow would be further improved. At the 95% threshold used in this study, a typical 15-minute SRS session could experience up to 45 seconds of cumulative beam-hold, which would not significantly impact patient or staff workflow. For the 6-minute mock SRS treatments in this study, the 95% threshold would correspond to ~18 seconds of beam-hold time. If higher positional accuracy is required, the tolerances can be tightened, with the trade-off of reduced DC and slightly longer treatment times. For example, from Table 1, the worst DC seen for 0.5 mm and 0.5° was 73% (volunteer #10); therefore, implementing this more accurate SRS delivery would result in ~97 seconds of extra delivery time.

A main advantage of the RHMC device is the continuous and incremental nature of head position adjustment throughout treatment delivery. These adjustments are activated at a low threshold of 0.2 mm and 0.2° and are much more accurate than manual intervention performed with the 1.0 mm and 1.0° tolerance level used for conventional frameless SGRT SRS.^31^ For manual intervention, there may be situations where the target can drift just under the manual intervention threshold for long periods of time, reducing the overall dosimetric accuracy. Such situations can be seen by the large areas above the curve for many of the non-RHMC cases in Figure 5.

Although the RHMC system performed well in terms of maintaining the target position below the clinically recommended tolerance, its performance was worse than that of the previous prototype.^20^ That prototype maintained volunteers’ head positions within 0.5 mm and 0.5° 99% of the time, whereas the current RHMC system did so 92% of the time. This decline in performance is attributable to the nonconstant frame rate of the SGRT system, in contrast to the constant frame rate of the previous prototype’s IR camera.^25^ The variable time delay of the SGRT system is extremely challenging to control because such delays cause linear phase shifts that limit the control bandwidth and affect closed-loop stability. To address this challenge, more sophisticated motion controller designs, such as those incorporating robust gain-scheduling feedback control, which can adapt to varying operating conditions and uncertainties, could be used.^35–38^ By designing for different parameter values and then interpolating or switching between them according to the measured parameter values, one can achieve a controller that can handle a wide range of scenarios. Nevertheless, the simplest and likely most robust solution would be to use an SGRT camera with a constant frame rate, which would likely improve the accuracy of the RHMC device head position control, based on previous work conducted with constant-frame-rate tracking cameras.^20^

Although masks can be used with the RHMC system (Fig. 1e), the primary goal of this work was to reduce invasiveness and enhance the clinical efficiency of SRS by eliminating frame and mask use. We thus created a universal frameless and maskless head holder that is not patient-specific and requires no setup beyond a patient’s lying supine and placing their head in it. The head holder’s 4-post design contributed to the achievement of excellent head motion stabilization; however, 1 volunteer had a smaller head that did not fully rest on all 4 posts, resulting in poor holder-to-robot coupling. To address this issue, a set of holders for different head sizes could be provided with the RHMC device, allowing the selection of the best-fitting head holder for each patient.

Given the lack of successful head motion stabilization for 1 volunteer with limited neck mobility in this study, we recommend the development of a simple RHMC suitability test to facilitate the clinical use of the system. For example, the RHMC system could be placed on the computed tomography scan couch during computed tomography scan simulation, and the robot could be commanded to perform a sequence of well-defined movements along various 6D axes to assess the ability to control the patient’s head motion.

Although we did not observe any change in the RHMC device accuracy at different couch angles, it is important to note that precautions must be taken when moving the couch while the RHMC device is enabled because couch movement can produce large apparent motion changes from the camera’s perspective. To prevent the robot from acting on these false head position deviations, it should be paused until the couch rotation is complete and the new couch angle has been registered (zeroed) using the camera.

UDP was used for SGRT-robot communication in this study. Although no communication error was observed, UDP is a connectionless protocol and does not check for missing or corrupt data packets. Given the high 6D position update frame rate of the SGRT camera (~10 fps), the slow speed of the robot (<1 mm/s), and the decoding and filtering of data packets by the robot’s motion controller, missing or corrupt data packets would not likely affect the system’s performance. However, in a version produced for clinical use, UDP could be replaced by the more robust transmission control protocol, which has a 2-way connection-based protocol that ensures that all data packets are delivered correctly.

## Conclusions

The RHMC device investigated in this study maintained human head motion within a clinical standard-of-care tolerance zone of <1.0 mm translationally and <1.0° rotationally for >95% of the treatment time in all suitable healthy volunteers without the use of a frame or mask. This device, which features a robot positioned beneath the patient’s shoulders and a superiorly extending head support, enabled full Linac gantry clearance and 360° radiation beam access—achieving this requirement for SRS for the first time. In addition to eliminating the need for frame or mask immobilization, the RHMC system has the potential to reduce patient setup and treatment times by minimizing manual interventions to correct out-of-tolerance positioning.

## Figures and Tables

**Table 1:** Comparison of the percentage of treatment time that the target remained within 1.0 mm and 1.0°, 1.0 mm and 0.5°, and 0.5 mm and 0.5° positional tolerance without (W/O RHMC) and with (W RHMC) RHMC across all 20 volunteers.

Vol#	1	2	3	4	5	6	7	8	9	10	11	12	13	14	15	16	17	18	19	20	Mean	Range
**Percent of time that target position is within 1.0 mm and 1.0° [%]**																						
W/O RHMC	61	93	77	23	16	9	100	88	24	99	100	100	99	100	21	97	100	100	71	96	73	9–100
W RHMC	100	100	100	100	99	100	100	97	99	98	100	100	97	99	96	100	100	96	66	10	99	96–100
**Percent of time that target position is within 1.0 mm and 0.5° [%]**																						
W/O RHMC	17	93	23	10	16	4	100	72	8	40	37	100	61	100	10	10	11	100	23	44	45	8–100
W RHMC	100	100	99	100	99	100	100	94	99	98	100	100	97	98	96	100	100	96	27	8	99	94–100
**Percent of time that target position is within 0.5 mm and 0.5° [%]**																						
W/O RHMC	0	60	8	10	13	2	100	64	8	40	37	100	61	99	10	10	9	99	23	34	41	0–100
W RHMC	100	99	98	98	93	100	100	88	94	73	85	96	79	91	82	100	93	85	27	5	92	73–100

*Abbreviations:* RHMC = robotic head motion compensation; W = with; W/O = without.

Data from excluded volunteers (#19 and #20) were not included in the calculations of the mean and range. Gray highlights cases where a 95% duty cycle was not achieved.

## Data Availability

Research data are stored in an institutional repository and will be shared uponrequest to the corresponding author.
